# Changes of direction during high-intensity intermittent runs: neuromuscular and metabolic responses

**DOI:** 10.1186/2052-1847-6-2

**Published:** 2014-01-13

**Authors:** Karim Hader, Alberto Mendez-Villanueva, Said Ahmaidi, Ben K Williams, Martin Buchheit

**Affiliations:** 1 Laboratory of Exercise Physiology and Rehabilitation”, EA 3300, Faculty of Sport Sciences, University of Picardie, Jules Verne, 80025 Amiens, France; 2 Sport Science Department, ASPIRE Academy for Sports Excellence, Doha, Qatar

**Keywords:** Cardiorespiratory responses, Neuromuscular adjustment, Selective activation, Knee stabilization

## Abstract

**Background:**

The ability to sustain brief high-intensity intermittent efforts (HIE) is meant to be a major attribute for performance in team sports. Adding changes of direction to HIE is believed to increase the specificity of training drills with respect to game demands. The aim of this study was to investigate the influence of 90°-changes of direction (COD) during HIE on metabolic and neuromuscular responses.

**Methods:**

Eleven male, team sport players (30.5 ± 3.6 y) performed randomly HIE without (straight-line, 2×[10× 22 m]) or with (2×[10× ~16.5 m]) two 90°-COD. To account for the time lost while changing direction, the distance for COD runs during HIE was individually adjusted using the ratio between straight-line and COD sprints. Players also performed 2 countermovement (CMJ) and 2 drop (DJ) jumps, during and post HIE. Pulmonary oxygen uptake (*V*O_2_), quadriceps and hamstring oxygenation, blood lactate concentration (Δ[La]_b_), electromyography amplitude (RMS) of eight lower limb muscles and rating of perceived exertion (RPE) were measured for each condition.

**Results:**

During HIE, CODs had no substantial effects on changes in *V*O_2_, oxygenation, CMJ and DJ performance and RPE (all differences in the changes rated as unclear). Conversely, compared with straight-line runs, COD-runs were associated with a possibly higher Δ[La]_b_ (+9.7 ± 10.4%, with chances for greater/similar/lower values of 57/42/0%) and either a lower (i.e., −11.9 ± 14.6%, 2/13/85 for semitendinosus and −8.5 ± 9.3%, 1/21/78 for lateral gastrocnemius) or equivalent decrease in electromyography amplitude.

**Conclusion:**

Adding two 90°-CODs on adjusted distance during two sets of HIE is likely to elicit equivalent decreases in CMJ and DJ height, and similar cardiorespiratory and perceptual responses, despite a lower average running speed. A fatigue-induced modification in lower limb control observed with CODs may have elicited a selective reduction of electromyography activity in hamstring muscles and may induce, in turn, a potential mechanical loss of knee stability. Therefore, changing direction during HIE, with adjusted COD running distances, might be an effective training practice 1) to manipulate some components of the acute physiological load of HIE, 2) to promote long-term COD-specific neuromuscular adaptations aimed at improving performance and knee joint stability.

## Background

The large majority of team sports (e.g., soccer, rugby, basketball, handball, Australian Football Rules) are characterized by a high number (>150) of brief (from 2 s to 10 s), high-intensity efforts which can take place at decisive moments of the match [1,2]. The ability to sustain these high-intensity intermittent efforts (HIE) throughout the entire match is a desirable physiological attribute to be competitive at the highest level [3]. In addition to these HIE, and many times superimposed to them, players perform more than 700 changes of direction (COD) per match [4]. The ability to COD while running at high-intensity has been recognized as an important factor for a successful participation in team sports [5]. Additionally, the speed associated with maximal oxygen uptake (v*V*O_2_max) has been suggested to be an important determinant of high-intensity intermittent exercise performance [6]. For a given absolute running speed, a high v*V*O_2_max may reduce the anaerobic contribution, thereby sparing glucose muscle stores, delaying fatigue and improving exercise tolerance. Moreover, while technique, straight-line speed and muscle qualities (i.e., strength, power) are factors that could influence the ability to change of direction [7], the impact of these factors on HIE performance including COD are still unclear.

To improve the ability to perform HIE, training programs typically include either intermittent supramaximal efforts (i.e., above v*V*O_2_max) [8] or repeated sprints [9,10]. However, since replicating game-specific high-intensity effort/recovery ratio is almost impossible given both the unpredictable nature of a game and the important position and individual playing style-related differences [11], coaches generally use simplified and pre-planned high-intensity runs in the field. These exercises differ, among other, in the distance, number and intensity of the runs, the duration and intensity of recovery, and the nature of the runs (e.g., the inclusion or not of COD, the distance of the shuttle). The introduction of COD (e.g., 15 s/15 s with shuttle runs [12], repeated shuttle-sprints [10]) is meant to increase the specificity of these training drills with respect to game demands (i.e., repeated turns at high speed). However, while our knowledge on the acute metabolic responses to straight-line HIE is improving (e.g., cardiorespiratory [13], muscle tissue oxygenation [14] responses), little is known on the physiological response to these more game-specific training drills including COD. Understanding theses acute physiological responses have important implications for specific training prescriptions in team sports [15].

Compared with straight-line HIE runs, high-intensity (but not all-out) 180°-COD runs may elicit higher heart rates (HR), blood lactate ([La]_b_) levels and rating of perceived exertion (RPE) [12]. During repeated sprints, introducing 180°-COD can also increase cardiorespiratory responses, but is unlikely to affect quadriceps deoxygenation levels and might surprisingly reduce the acute neuromuscular load (as inferred by a lower running speed decrement compared with straight line runs) [16]. However, since in these latter studies, speed [12] or distance [16] was not adjusted for time lost while COD, the respective effect of COD *per se* could not be examined. When accounting specifically for COD abilities (i.e., accounting for the time lost while changing direction), repeated straight-line sprints appeared physiologically and perceptually more demanding than sprints with 90° and 135°-COD [17]. However, in this latter study [17], measures were limited to HR and RPE, which prevent from drawing definitive conclusions. Additional measures such as cardiorespiratory and muscle deoxygenation demands might enable a more comprehensive understanding of the acute physiological responses to HIE with COD.

Finally, lower limb muscle activation has also been reported to increase during COD tasks compared with straight-line runs [18]. This augmented muscle activity is believed to help maintaining knee join stabilization during COD tasks in response to the varus/valgus and internal/external rotation moments of the knee [18]. During repeated high-intensity runs such as during HIE, fatigue typically appears, together with impaired neuromuscular function [19]. Fatigue may also promote extreme lower biomechanics stemming from inadequate active joint stabilization via a suboptimal muscle activation strategy [20,21]. This fatigue-induced suboptimal neuromuscular function strategy is thought to place an individual at a greater risk for lower limb injuries (e.g., anterior cruciate ligament) [20]. For these reasons, during HIE including COD, the musculature surrounding the knee and its respective neural adjustments is critical with respect to injury risk and prevention [22]. However, the specific adjustments in muscle activity patterns during HIE practices, as presently observed in the field (e.g., training [10] or games [4]) are still unknown. A better understanding of muscle activation adjustments with COD is useful to design HIE protocols including COD and, in turn, manipulate the mechanical factors affecting lower limb stability. This could be helpful to either reduce acute injury risk during such HIE training sessions, or promote long-term COD-specific neuromuscular adaptations (i.e., performance enhancement and injury prevention strategies).

Therefore, the purpose of this study was to compare changes in metabolic (i.e., cardiorespiratory, [La]_b_, muscle deoxygenation), neuromuscular (i.e., jumping ability and muscle electromyography activity) and perceptual responses to straight-line and COD HIE runs, while accounting for COD abilities [17]. We expected that the greater absolute speed reached during the straight-line condition [17] would induce greater muscle activation, and, in turn, greater overall physiological load and an exacerbated impairment in neuromuscular performance.

## Methods

### Subjects

We recruited eleven amateur team sport players (mean ± SD: 30.5 ± 3.6 y, 81 ± 6 kg, 180 ± 6 cm) for this study. They were all involved (4.9 ± 2.7 h.wk^-1^) in soccer (n = 3), handball (n = 2) or Australian Rules football (n = 6) and had no history or clinical signs of cardiovascular or pulmonary diseases. Players were not currently taking prescribed medications and presented with normal blood pressure levels and electrocardiographic patterns. All players gave voluntary written consent to participate in the experiment. The study conformed to the recommendations of the Declaration of Helsinki and was approved by the local Review Board for use of Human Subjects.

### Experimental overview

**Figure 1:**
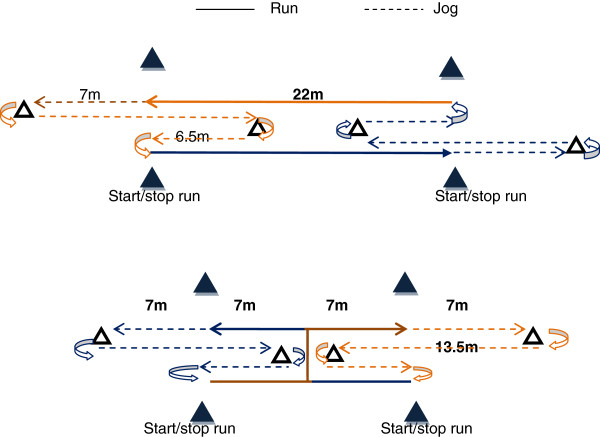
Schematic of the straight-line (upper panel) and two 90°-COD (lower panel) runs.

Each player was tested on four occasions, separated by at least 48 h. During the first session, players performed an incremental running test (Vam-Eval) to determine maximal oxygen uptake (*V*O_2_max) and the associated velocity (v*V*O_2_max). During the second session, players performed two 40-m sprints to determine their maximal sprinting speed and 3 maximal 22-m sprints with two 90°-COD to assess their COD speed. During sessions 3 and 4, in a randomized order, participants performed a HIE including runs either in straight-line or with two 90°-COD. To compensate for the time lost while changing direction and accurately examine the specific impact of CODs on performance, physiological and perceptual responses to HIE, the distance of the runs during the HIE including CODs was adjusted (reduced) on an individual basis [17] (Figure 1).

Cardiorespiratory variables, electromyography for eight lower limb muscles, vastus lateralis (VL) and biceps femoris (BF) oxygenation levels (near-infrared spectroscopy, NIRS), [La]_b_ and RPE (0–10 on Borg’s scale) were collected for all tests. Players also performed 2 countermovement jumps (CMJ) and 2 drop jumps (DJ) prior, at mid and post HIE. All tests were performed on an indoor synthetic track where ambient temperature ranged from 18 to 22°C. Subjects were told not to perform exercise on the day prior to a test, and to consume their last (caffeine free) meal at least 3 h before the scheduled test time.

### Exercise protocols

#### Incremental running test

A modified version of the University of Montreal Track Test ([23] (i.e., Vam-Eval) was used on an indoor synthetic 400-m track. The Vam-Eval is very similar to the University of Montreal Track Test, i.e., same speed increments. The only difference between the two tests is the distance between the cones placed along the athletic track (i.e., 20 (Vam-Eval) vs. 50 (University of Montreal Track Test) m), which renders the Vam-Eval easier to administer. The running speed started at 8 km.h^-1^ with consecutive speed increments of 0.5 km.h^-1^ each minute until exhaustion. Running pace was governed by a pre-recorded beep that sounded at appropriate intervals to adjust running speed as they passed close to visual marks, every 20 m, along the track. Players were instructed to complete as many “stages” as possible, and the test ended when the player could no longer maintain the required running speed (i.e. when players were unable to reach the marks the moment the audio signaled on 3 consecutive occasions). The reliability of v*V*O_2_max is good in moderately-trained middle and long distance runners, with a typical error, expressed as a coefficient of variation (CV) of 3% [24].

#### Speed test

The speed tests were preceded by a supervised and standardized warm-up consisting of 5 min of running at ~10 km.h^-1^, 3 min of athletic drills (e.g., skipping, high knee runs), 5 short bursts of progressive accelerations on the track and 2 maximal 15-m straight-line sprints. All players performed 1) two maximal 40-m sprints during which 10-m, 20-m, 22-m and 30-m split times were recorded using dual-beam electronic timing gates (Swift Performance Equipment, Lismore, Australia), 2) two maximal 22-m sprints with two 90°-COD. Players performed the CODs between 2 sticks (height 1.5 m) placed vertically with an interdistance of 1.2 m. All sprints were interspersed by 90 s of passive recovery. The players commenced each sprint from a standing start with their front foot 0.10 m behind the first timing gate and were instructed to sprint as fast as possible. They started when ready, thus eliminating reaction time. Maximal sprint speed (MSS) was defined as the fastest 10-m split time over the same maximal 40-m sprint [25]. The reliability of MSS was assessed prior to the present study in a group of 65 young soccer players: the typical error, expressed as a CV, was 1.4%, 90% confidence limits (1.2; 1.6).

#### Jumping ability

Lower limb explosive strength was inferred via jumping height (cm) in vertical CMJ and DJ measured by a force plate (Kistler Instruments, Amherst, Massachusetts). Each type of jump was repeated twice before, between and after the 2 running sets of HIE. For both CMJ and DJ, the average jumping height over the two trials at each time point was retained for analysis. The participants were instructed to keep their hands on their hips during both CMJ and DJ. For CMJ, the players were instructed to dip to their optimal depth from a standing position and immediately jump for maximum height. CMJ were performed in a continuous movement with no pause between downward and upward phases. DJ were executed from a 36-cm box without any leg flexion. Players were requested to minimize ground contact time and to jump as high as possible. Each trial was validated by visual inspection. Three scores were calculated for the sequences: the mean jumping height and the percent jumping height decrement (% Dec;%), as adapted from Spencer, Fitzsimons et al. [26]:

$$\left[\left(\mathrm{mean}\;\mathrm{jumping}\;\mathrm{height}/\mathrm{pre}-\mathrm{test}\;\mathrm{jumping}\;\mathrm{height}\times 100\right)-100\right].$$

#### High-intensity efforts

Both straight-line and COD HIE were preceded by a supervised and standardized warm up consisting of 5 min running at ~10 km.h^-1^. In addition, the participants performed 3 min of athletic drills (e. g., skipping, high knee runs), 5 short bursts of progressive accelerations on the track and two 22-m straight-line sprints. Following jumps, the subjects completed the HIE protocol consisting of 10 repeated 4-s straight-line or COD-runs, departing every 16 s. To account for the time lost while changing direction, the distance for COD runs during HIE was individually adjusted [17] using the ratio [27] between the 22-m straight-line and COD-sprints as follows:

$$\begin{array}{l} \mathrm{Adjusted}\;\mathrm{HIE}\;\mathrm{COD}\;\mathrm{distance}\left(m\right)\\ \;=\mathrm{Straight}-\mathrm{sprint}\;\mathrm{time}\left(s\right)\times 22/22-\mathrm{mCOD}-\mathrm{sprint}\;\mathrm{time}\left(s\right) \end{array}$$

This 10-run set was repeated twice (2 sets), interspersed with 2 min 20 s. This effort sequence was chosen based on the high-intensity intermittent effort profile of the majority of team sport matches (e.g., 2.2 s/18 s in soccer [28]) and the work/recovery ratio of the most intense 5 min period (i.e., 2.2 s/13 s [29-31]. Between each 4-s run, subjects performed the 16-s active recovery running at 6 km.h^-1^. An audio feedback (i.e., time countdown) was given to the subjects so that they maintained the required running speed. Three seconds prior to the commencement of each run, subjects were asked to assume the ready position and await the start signal. They were instructed to complete all runs within the allocated time (i.e., 4 s), and strong verbal encouragement was provided during all runs. If a player was not able to perform two consecutive runs in the allocated time, the test was considered as finished. Nevertheless, all players managed to complete the 2 sets.

### Data collection and analyses

#### Cardiorespiratory measures

Respiratory gas exchange was measured using an automated, portable, breath-by-breath system (Oxycon Pro, Carefusion GmbH, Hoechberg, Germany) during all tests. Before each test, the O_2_ and CO_2_ analysis systems were calibrated as recommended by the manufacturer. Cardiorespiratory values were averaged over 5-s periods. During the incremental test, *V*O_2_max was defined as the highest *V*O_2_ values attained in 30 s and v*V*O_2_max as the lowest running velocity maintained for at least one minute that elicited *V*O_2_max [32].

#### Near-infrared spectroscopy measurements

The portable NIRS apparatus (Portamon, Artinis, Medical System, Zetten, The Netherlands) used in this study is a 2-wavelength continuous wave system, which simultaneously uses the modified Beer–Lambert and spatially resolved spectroscopy methods. The procedure used to collect data was the same as described previously with a similar portable device [16]. Changes in tissue oxyhemoglobin (HbO_2_) and deoxyhemoglobin (HHb) were measured using the differences in absorption characteristics of light at 750 and 850 nm. The difference between HbO_2_ and HHb (Hbdiff = (HbO_2_-HHb)/2) was also calculated. Given the uncertainty of the proton pathlength at rest and during exercise, we used an arbitrary value for the differential pathlength of 3.83. The values for Hbdiff were reported as a change from baseline (30 s averaging before each test) in micromolar units (μM, HbO_2_, HHb and Hbdiff) [33]. The use of Hbdiff was considered since it has been shown to be a relevant alternative to HHb when total hemoglobin (tHb) is not constant; muscle oxygen consumption estimated from Hbdiff being more reliable than values estimated from the other NIRS variables [34]. The reliability of the NIRS technique during high-intensity runs is acceptable, with coefficients of variation ranging from 3 to 35%. The sensitivity of the NIRS technique to exercise intensity is remarkable, as evidenced by the substantial differences in muscle oxygenation observed when comparing straight-line vs. COD runs [35]. Moreover, we paid great attention to probe replacement. With the portable device used, firmly attached to the body, there are no moving optical fibers that could cause signal disturbance. NIRS probes were positioned on the vastus lateralis and biceps femoris muscles of the dominant leg (i.e., the kicking leg) used when changing direction, approximately 10 cm from the knee joint and along the vertical axis of the thigh. A surgical marker was used to mark the probe placement for accurate repositioning. The probe and the skin were covered with black tape to prevent contamination from ambient light. During all tests, the NIRS system was connected to a personal computer by Bluetooth for data acquisition (10 Hz), analogue-to-digital conversion, and subsequent analysis.

#### Blood lactate measurement

Just after the warm-up preceding HIE runs and immediately at the end of each set, a fingertip blood sample (5 μL) was collected and blood lactate concentration was determined (Lactate Pro, Arkray Inc, Japan). The accuracy of the analyzer was checked before each test using standards. The suitability and reproducibility of this analyzer has been previously established throughout the physiological range of 1.0 – 18.0 mmol.L^-1^[36].

#### Electromyography measurement

During both conditions, electromyography (EMG) data were collected from the dominant leg, using an eight channel Datalog EMG system (Biometrics DataLOG P3X8, Gwent, UK). The contracted muscle belly of the VL, BF, gastrocnemius medialis (MG) and lateralis (LG), vastus medialis (VM), semitendinosus (ST), adductor longus (AL) and gluteus medius (GM) were identified. A surgical marker was used to mark the electrodes placement for accurate repositioning, in accordance with the Surface EMG for Non-invasive Assessment of Muscles recommendations (SENIAM) [37]. Before placing the electrodes, the overlying skin was carefully prepared. The hair was shaved, and the skin was lightly abraded to remove the outer layer of epidermal cells and thoroughly cleansed with alcohol to reduce the skin–electrode interface impedance. Biometrics SX230 active (Ag/AgCl) electrodes separated by 2 cm were carefully taped to the belly of each muscle, parallel to the muscle fibers, using hypoallergenic adhesive tape and cotton wool swabs to minimize sweat induced interference. A passive reference electrode (Biometrics R300) was placed on the pisiform bone of the wrist, with its wiring passed through participants’ t-shirt to allow free running movements and to restrict its impedance. The EMG device was secured and fixed to a waist belt. To prevent movement artifact, wires between the electrodes and the device were secured to the skin with adhesive tape and leads braided to minimize electromagnetically induced interference. Signals were sampled at 1000 Hz, amplified (1000×), band-pass filtered (20–450 Hz), and stored for offline analysis on a 512 MB MMC flashcard (Biometrics DataLOG P3X8; Gwent, UK). Data were imported from the Biometrics unit in 1-ms increments into Spike 2 version 5 (Cambridge Electronics Design, Cambridge, UK) and saved for offline analysis. The data were smoothed using route mean squared analysis (RMS), which was calculated for a 50-ms window.

EMG data (μV) were calculated for each step (active contraction). Since Arsenault et Winter [38] showed that a minimum of three strides of EMG data per subject provided information as reliable as that obtained from twelve strides during gait trials, we analyzed five strides taken in the middle of the run, with similar peak amplitudes [39]. Onset and offset of muscle activity were determined as a deviation greater than two standard deviations from the mean of three 50-ms windows of inactivity. The fastest 22-m straight-line sprint was also analyzed by isolating five peak amplitude contractions from the middle of the sprint. The resultant mean amplitudes were averaged and used for normalization, i.e., the EMG data from HIE runs were expressed as a percentage of the EMG measured during the fastest straight-line-sprint [39].

### Statistical analyses

Data in the text and figures are presented as means ± SD. All data were log-transformed for analysis to reduce bias arising from non-uniformity error and then analysed for practical significance using magnitude-based inferences [40]. We used this qualitative approach because traditional statistical approaches often do not indicate the magnitude of an effect, which is typically more relevant to athletic performance than any statistically significant effect. For between-condition comparisons, the chance that the true (unknown) values for COD runs were *higher* (i.e., greater than the smallest practically important difference, or the smallest worthwhile [difference] change, SWC [0.2 multiplied by the between-subject standard deviation, based on Cohen’s ES principle]), *similar* or *lower* was calculated using a specifically-designed Excel spreadsheet [41]. Quantitative chances of better/greater or worse/smaller performance or physiological and perceptual responses were assessed qualitatively as follows: ≤1%, almost certainly not; >1-5%, very unlikely; >5-25%, unlikely; >25-75%, possible; >75-95%, likely; >95-99, very likely; >99%, almost certain. If the chance of having greater/better or detrimental/lower performances (or responses) were both >5%, the true difference was assessed as unclear.

## Results

### Sprint distance adjustment

The straight-line-sprint time/COD-sprint time ratio was 0.75 ± 0.02. Running distance with COD was therefore reduced by a factor of 0.75 in average, i.e., adjusted from 22 to 16.5 ± 0.4 m. HIE averaged speed in straight-line represented 85.6 ± 2.3%MSS and 131.1 ± 12.9%v*V*O_2_max. HIE with COD was performed at an average speed of 50.8 ± 1.7%MSS and 98.7 ± 9.7%v*V*O_2_max.

### Metabolic responses during HIE, with or without COD

**Figure 2:**
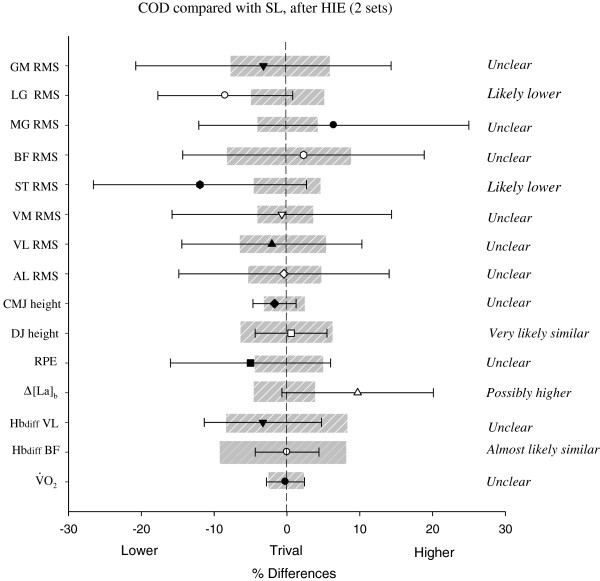
**Changes in oxygen uptake (*V*O**_**2 **_**expressed as a percentage of maximal oxygen uptake), vastus lateralis (Hbdiff VL) and biceps femoris deoxygenation (Hbdiff BF), changes in blood lactate concentration (Δ[La]**_**b**_**), rating of perceived exertion (RPE), drop jump (DJ) and countermovement jumping (CMJ) heights and EMG amplitude (RMS) in 8 muscles during and after high-intensity intermittent efforts (HIE), with (COD) or without (i.e., straight-line, SL) changes of direction.** AL: adductor longus; VL: vastus lateralis; VM: vastus medialis; ST: musculus semitendinosus; BF: biceps femoris; MG: medial gastrocnemius; LG: lateral gastrocnemius; GL: gluteus medius; n = 11.

**Figure 3:**
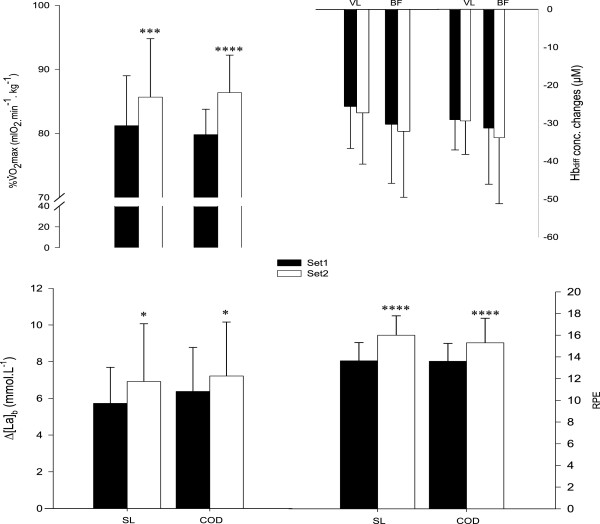
**Changes in oxygen uptake (*V*O**_**2, **_**expressed as a percentage of maximal oxygen uptake), vastus lateralis (VL) and biceps femoris (BF) deoxygenation (Hbdiff), changes in blood lactate concentration (Δ[La]**_**b**_**) and rating of perceived exertion (RPE) during and after each set of high-intensity intermittent efforts (HIE), with (COD) or without (i.e., straight-line, SL) changes of direction.** VL: vastus lateralis muscle; BF: biceps femoris muscle; *: possible within-condition difference vs. Set1; ***: very likely within-condition difference; ****: most likely within-condition difference; n = 11.

**Figure 4:**
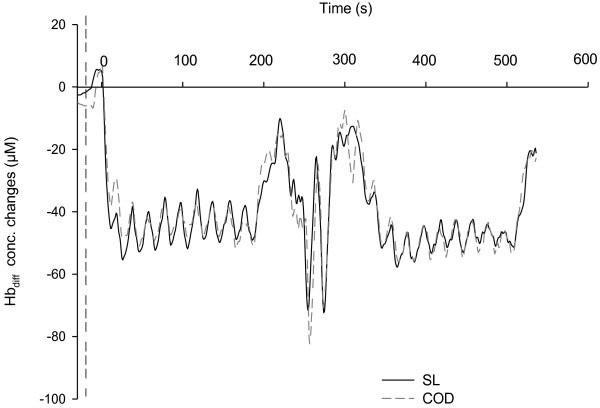
Changes in vastus lateralis (VL) deoxygenation (Hbdiff) in one representative subject during straight-line (SL) and changes of direction (COD) protocols.

Compared with the 1st set, there was a very likely (straight-line, +5.4 ± 2.5%, with chances for greater/similar/lower values of 99/1/0, respectively) to almost certain (COD, +8.1 ± 2.0%, 100/0/0) increase in *V*O_2_ during the 2nd set. There was however, no between-condition difference in *V*O_2_ (difference rated as unclear, Figure 2). As presented in Figure 3, mean Hbdiff values for VL and BF muscles were likely similar between sets in both conditions (differences rated as unclear, Figure 2). Figure 4 shows the typical changes in Hbdiff in a representative player.

Compared with the 1st set, Δ[La]_b_ was possibly greater after the 2nd set during straight-line (+11.5 ± 14.1%, 60/39/1) and COD (+8.6 ± 8.6%, 56/44/0) (Figure 3). Compared with straight-line, the average [La]_b_ throughout the 2 HIE sets was possibly higher with COD (+9.7 ± 10.4%, 57/42/0), Figure 3).

### Jumping performance during and after HIE, with or without COD

**Figure 5:**
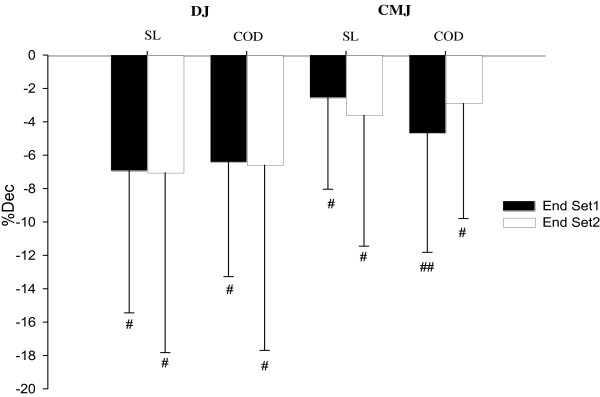
**Percent jumping height decrement (% Dec) during and after each set of high-intensity intermittent efforts (HIE), while performed with (COD) or without (i.e., straight-line, SL) changes of direction.** CMJ: countermovement jump; DJ: drop jump; COD: change of direction. #: possible within condition difference vs. pre-test jump, ##: likely within condition difference. n = 11.

Prior to testing, mean CMJ height was 37.3 ± 4.5 and 36.9 ± 4.4 cm for straight-line and COD conditions, respectively, with no difference between the 2 conditions. Compared with baseline, CMJ height was at least possibly decreased by 2.5 ± 5.5% (straight-line, with chances for greater/similar/lower values of 1/49/50, respectively) and 4.7 ± 7.2% (COD, 1/11/88) after the 1st set, and by 3.6 ± 7.9 (straight-line, 1/30/69) and 2.9 ± 6.9% (COD, 1/25/74) after the second set (Figure 5). Prior to testing, mean DJ height was 23.7 ± 6.2 and 23.8 ± 6.4 cm for straight-line and COD conditions, respectively, with no difference between the 2 conditions. Compared with baseline, DJ height was decreased by 6.9 ± 8.5% (straight-line, 0/36/64, respectively) and 6.4 ± 6.9% (COD, 0/48/52) after the 1st set, and by 7.0 ± 10.8% (straight-line, 0/35/65) and 6.6 ± 11.1% (COD, 0/42/58) after the second set (Figure 5). After the 2nd set, the between-condition difference in the changes from baseline was unclear for CMJ and very likely similar for DJ (Figure 2).

### EMG activity during HIE, with or without COD

**Figure 6:**
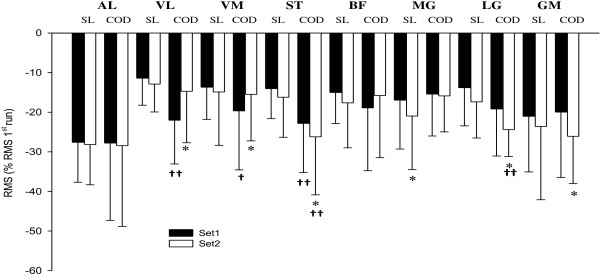
**Changes in electromyography amplitude (RMS) for 8 muscles during and after high-intensity intermittent efforts, with (COD) or without (i.e., straight-line, SL) changes of direction.** *: possible within-condition difference vs. 1st set; †: possible difference vs. SL; ††: likely different. AL: adductor longus; VL: vastus lateralis; VM: vastus medialis; ST: musculus semitendinosus; BF: biceps femoris; MG: medial gastrocnemius; LG: lateral gastrocnemius; GL: gluteus medius. SL: straight-line; COD: change of direction; n = 11.

The changes in RMS at the end of both HIE sets are displayed in Figure 6. For both conditions and for all muscles, RMS was very likely decreased after each set.

During straight-line, compared with the end of the 1st set, MG RMS was further decreased after the 2nd set (−4.8 ± 3.7%, 0/37/63). There was no further change in RMS after the 2nd set for the other muscles (all changes rated as unclear or similar).

With COD, compared with the end of the 1st set, ST RMS (−4.4 ± 8.3%, 5/38/57), LG (−6.4 ± 9.1%, 2/39/59) and GL (−7.6 ± 8.3%, 0/46/53) were further decreased after the 2nd set. In contrast, VL (+9.4 ± 15.2%, 50/48/2) and VM (+5.2 ± 6.9%, 61/38/2) RMS were possibly greater after the 2nd set compared with the end of the 1st set.

Between-condition comparisons in the changes in EMG activity are presented in Figure 2. Compared with straight-line, changes in VL RMS (−12 ± 11.5%, 0/15/84) and ST RMS (−10.2 ± 12.2%, 2/16/83) were likely lower, and these in VM RMS (−6.9 ± 11.8%, 5/26/69) possibly lower with COD after 1st set. There was no between-condition difference for the other muscles (all differences rated as unclear or similar).

After the 2nd set, compared with straight-line, ST RMS (−11.9 ± 14.6%, 2/13/85) and LG RMS (−8.5 ± 9.3%, 1/21/78) were likely lower with COD. There was no between-condition difference for the other muscles (all differences rated as unclear or similar).

### Perceptual responses during HIE, with or without COD

As presented in Figure 3, RPE was almost certainly greater after the 2nd set compared with the 1st, for both conditions. There was no between-condition difference for the average RPE values, throughout the 2 HIE sets (differences rated as unclear, Figure 2).

## Discussion

In this study, we examined metabolic, jumping performance, neuromuscular and perceptual responses to repeated brief straight-line or 90°-COD high-intensity runs. To account for the time lost while changing direction, the distance for COD runs during HIE was adjusted using the ratio between the straight-line and COD-22-m sprints [17]. This allowed us to investigate for the first time the effect of COD *per se* on the aforementioned variables during HIE. The main results were as follows: 1) despite a lower average running speed during HIE, CODs are likely to elicit an equivalent *V*O_2_, quadriceps and hamstring deoxyenation, perceived exertion and changes in jumping performance, 2) CODs elicit a possibly higher blood lactate accumulation compared with straight-line, 3) CODs are associated with an either equivalent or greater decrease in RMS for all muscles, including hamstrings, compared with straight-line.

### Metabolic responses

Despite the brief duration of each running effort, HIE was characterized by a high aerobic demand for both conditions (i.e., 85.7 ± 9.1%*V*O_2_max in straight-line and 86.4 ± 5.8%*V*O_2_max with COD during the 2nd set). This could be explained by the fact that, even during very short (<10 s), repeated, high-intensity efforts, the aerobic ATP provision progressively increases and can contribute to a great extent to the total energy supply during the final repetition [42]. After the first set of HIE with COD, *V*O_2_ responses were equivalent to those of repeated sprints with one, 180°-COD (i.e., 80 ± 4%*V*O_2_max during HIE vs. 80.5 ± 10.3%*V*O_2_max during repeated sprints) [16]. Our main finding was that there were no differences in *V*O_2_ responses during HIE between conditions (Figure 2). While this result contrasts with the higher *V*O_2_ reported by Buchheit et al. [16], COD sprints were also associated with a longer exercise time (i.e.,~30%) in this later study, which was likely to increase the overall metabolic demands independently of COD. With distances adjusted as in the present study, the possible extra cost of O_2_ related to CODs could have been balanced with the likely lower O_2_ demand in response to the ~25% lower average running speed reached in comparison with straight-line (i.e., ~98%v*V*O_2_max with COD vs. ~131%v*V*O_2_max in straight-line). Additionally, an alteration of the locomotion-ventilation coupling, as a result of changes in stride patterns and velocity, could have counterbalanced the lower running speed in the COD condition [43].

The high pulmonary *V*O_2_ demand was associated, for both conditions, with a rapid increase in quadriceps and hamstring deoxygenation at the start of each set, followed by a plateau throughout the runs (Figure 4). A similar plateau was reported during repeated cycling-sprints of 6 s interspersed with 30 s of passive rest [44,45] and could be interpreted as an evidence of maximal O_2_ extraction [44]. Interestingly, muscle deoxygenation remained similar during the second set despite a substantial increase in pulmonary *V*O_2_ (Figure 3), confirming that maximal extraction may have already been reached during the first set. We observed no differences in deoxygenation levels between conditions (Figure 2), which is in agreement with the study of Buchheit et al. [16], where quadriceps deoxygenation levels were similar for repeated (all-out) sprints performed with or without COD (25 vs. 2 × 12.5 m sprints) (despite the differences in running times). With the limitation of NIRS to monitor the actual muscle O_2_ demands, present results suggest that HIE with two 90°-COD and a ~25% lower average running speed, elicits, in comparison with straight-line runs, an equivalent modification of the local O_2_ uptake/delivery ratio, which is in agreement with the systemic *V*O_2_ measures. The extrapolation of this findings to the overall lower musculature should however be viewed with caution given the large heterogeneity of muscle (de)oxygenation within the same muscle [46].

Compared with straight-line, CODs were associated with a possibly greater blood lactate accumulation during HIE (Figure 2). Present results are in agreement with previous data collected during HIE runs with COD (i.e., 10s/10s with 180°-COD), but where the time lost while changing direction was not taken into account [12]. The higher blood lactate values reported in this later study were therefore not surprising since in the COD condition players had to run at a faster running speed to compensate for the time lost during COD. In contrast, when accounting for the time lost when COD and, hence, isolating the effect of COD *per se* on blood lactate responses, a very likely lower [La]_b_ was reported during repeated sprints with 90°-COD compared with straight-line [17]. The faster running speed used in this later study (i.e., all out sprints [17] vs. supramaximal (~130%*V*O_2_max), but not all-out runs as employed in the present study), might have overpowered the possible impact of the CODs during sprints (i.e., decelerations, stops and [re]accelerations) on blood lactate accumulation, leading in turn, to a higher physiological load [17].

### Jumping performance, neuromuscular and perceptual responses

We observed substantial reductions in jumping performance at the end of each HIE set for both conditions (Figure 5), indicating an acute decrease in the ability to generate explosive muscle contractions. This is consistent with the substantial decrements in CMJ height reported after repeated-sprint sequences in team sport players (−8% after 6, 25-m sprints, [35]). In the present study, the reduction in both DJ and CMJ performance was, however, similar for the two running conditions (Figure 2), suggesting comparable impairments in the explosive force production capabilities despite the substantially lower average speed attained during the COD trial.

Despite the similar reductions in explosive force production, the straight-line and COD tasks appear to involve different control strategies, as evidenced by the different adjustments in EMG activity during the two tasks (Figure 6). CODs led to likely (i.e., vastus lateralis and semitendinosus at the end of the 1st set; semitendinosus and gastrocnemius lateralis at the end of the 2nd set) and possibly (i.e., vastus medialis at the end of the 1st set) greater decrements in RMS compared with the straight-line condition (Figure 6). For the other muscles, there were no substantial differences in the decrease in RMS between conditions. While caution should be applied when inferring motor control strategies from EMG [47] and while raw RMS values can be affect by change in sarcolemmal excitability independently of actual muscle activation levels [48], the EMG data were expressed as a percentage of the EMG measured during the fastest straight-line-sprint [39], which partially compensate for these limitations. The overall greater rate of decrease in the surface EMG activity observed (i.e., RMS) suggests that the net inhibition of the motor neuron pools increased at a faster rate during the COD trial. Muscle activity (i.e., quadriceps, hamstring and gastrocnemius muscles) during COD tasks has been reported to be greater than during straight-line, being this increased muscle activation directed to stabilize the knee as it experiences larger valgus and rotation moments [18]. In this regard, hamstring muscles have been postulated to have a prominent role in providing stability to the knee joint complex [49,50]. On the other hand, a fatigue-induced reduction in neuromuscular activity of the hamstring muscles has been reported to be associated with a mechanical loss of knee stability [51], which may reduce the anterior cruciate ligament protection during COD movements [52]. Thus, the substantial reduction in motor unit activity in the semitendinosus muscle during the HIE with COD (Figure 6) might have induced larger valgus knee moments [53] or decreased joint stability [54]. Indeed, semitendinosus activity is believed to be important to compress the medial knee joint, to probably limit the risk of excessive dynamic valgus load of the knee joint and thereby reduce stress on the anterior cruciate ligament [55]. A selective fatigue-induced reduction in hamstring EMG activity has been reported during side cutting actions, in response to a simulated handball match play [52]. This reduction in semitendinosus EMG activity observed in the present study could potentially represent a strategy to maximize mechanical efficiency at the knee joint [52]. Such performance-enhancing strategy might explain the finding of an unchanged COD performance (subjects were able to complete both the straight-line and COD tasks as prescribed) despite the substantial decrease in explosive force capabilities (i.e., CMJ and DJ). Therefore, in the present study, the fatigue-induced decrease in semitendinosus EMG activity during CODs might optimize performance but, combined with an increase of vastus lateralis EMG activity during the 2nd set of HIE, might also represent an impaired motor pattern for optimal knee joint stabilization [55]. Potential candidates mechanisms for this reduced EMG activity during the COD task include either disfacilitation of the motor neuron pool due to a progressive decline in muscle spindle responsiveness [51,56], or presynaptic inhibition of Ia afferent feedback [57,58].

Finally, the addition of CODs did not modify RPE responses compared with straight-line HIE (Figure 2). These results contrast with the data reported by Buchheit et al. [17], where very likely lower RPE responses were observed during repeated (and time-adjusted) sprints with 90°-COD, compared with straight-line. As discussed above, differences in peak running speed between the protocols may explain these inconsistencies. We can also speculate that COD-related changes in muscle tensions are more marked at full (all-out) [17] than supramaximal (~130%*V*O_2_max) speed as in the present study, which may differently impact RPE responses.

### Practical applications

In the present study, we provide for the first time detailed information for coaches and strength and conditioning coaches on the physiological and perceptual responses to HIE sequences, which partially replicate both the most intense periods of a soccer game [28,29] and common training practices [8,9].

Present and previous [17] data suggest that coaches can adjust COD running distances (e.g., accounting for individual losses in speed while COD) and intensities (e.g., all out sprints vs. supramaximal runs) to manipulate both the acute neuromuscular and glycolytic load during HIE, in accordance to their sessions goal (e.g., more systemic or peripheral load) and training periodization. Coaches and strength and conditioning coaches should also be aware that the inclusion of CODs during HIE can be associated with an acute selective decrease in hamstrings muscle activity, which might compromise optimal knee joint stabilization and, in turn, increase the acute risk of injury. In contrast, when these sessions are appropriately implemented within the training cycle, these solicitations could promote long-term COD-specific neuromuscular adaptations aimed at improving performance and preventing potential injuries. Finally, the fact that RPE was not different between conditions despite the observed different glycolytic, neuromuscular and mechanical demands highlight the limitation of the session-RPE method [59] to quantify training load in team sports. This confirms previous on-field observations [60] suggesting that different motion patterns associated with clearly different neuromuscular demands (e.g., with or without COD, with or without the ball or opponents) may have similar perceptual (and cardiorespiratory) loads.

## Conclusion

In conclusion, while isolating the actual physiological and perceptual impact of COD during HIE, we observed that the addition of two 90°-COD is likely to slightly increase blood lactate accumulation and elicit a greater reduction in semitendinosus and gastrocnemius lateralis muscles EMG activity. In contrast, compared with straight-line runs, COD runs with time-adjusted distances induced similar impairment of lower limb explosive force production capacities (i.e., jumping performances), and equivalent cardio-respiratory and perceptual responses. Therefore, present results suggest that changing direction during HIE, with adjusted COD running distances, might be an effective training practice to manipulate some components of the acute physiological load of HIE. Despite a ~25% lower average running speed during HIE with COD, hamstring activation was substantially reduced, thereby potentially inducing a mechanical loss of knee stability. To further optimize training prescription, it would be interesting to investigate both the mechanism of the neuromuscular adjustments and the impact of other COD-angles and running distances on the observed variables.

## Competing interests

The authors declare that they have no competing interests.

## Authors’ contributions

KH participated in the study design, conducted the experiment, analyzed the data and drafted the manuscript, AM participated in the study design and provided critical comments on the manuscript, SA provided critical comments, BW participated in data collection and provided critical comments on the manuscript and MB participated in the study design, analyzed the data, participated in data collection and provided critical comments on the manuscript. All authors read and approved the final manuscript.

## Pre-publication history

The pre-publication history for this paper can be accessed here:

http://www.biomedcentral.com/2052-1847/6/2/prepub
